# Designing a Comprehensive Pattern of Structural-Psychological Empowerment for Employees of Medical Sciences Universities

**DOI:** 10.31661/gmj.v8i0.1486

**Published:** 2019-03-30

**Authors:** Nayereh Sadat Roohollahi, Iravan Masoudi Asl, Somayeh Hessam, Mahmoud Mahmodi

**Affiliations:** ^1^Department of Health Services Management, South Tehran Branch Islamic Azad University, Tehran, Iran; ^2^Department of Health Services Management, School of Health Management and Information Sciences, Iran University of Medical Sciences, Tehran, Iran; ^3^Department of Epidemiology, Tehran University of Medical Science, Tehran, Iran

**Keywords:** Pattern, Empowerment, Employee, University

## Abstract

**Background:**

The concept of empowerment requires the abandonment of traditional models. The need to design and develop employee empowerment patterns has been emphasized in several studies. The present study aims to design a comprehensive structural-psychological empowerment pattern for employees of medical sciences universities.

**Materials and Methods:**

Our exploratory research was conducted on 410 employees of medical universities of Tehran, Iran, and Islamic Azad University. Firstly, a primary pattern was designed according to a review of available literature, texts, patterns, and tools. Then, the psychometric analysis was done using validation (face validity, content validity, construct validity, factor validity) and reliability (internal consistency and stability). Lastly, the final pattern was introduced after having been approved by experts. Data were analyzed using SPSS version 24 and AMOS made by USA IBM software. P<0.05 was considered as the significance level.

**Results:**

Based on our study, 83.9% of participants were holders of bachelor’s degrees or higher degrees. The results of validation (face, content, structure, and confirmation validity) and reliability (internal consistency [α=0.90] and stability [0.91]) showed that the structural-psychological empowerment pattern was appropriate, which was validated with 31 items and 8 domains. The scope of this pattern included resources, self-sufficiency, competence, support, effectiveness, and opportunity, significance, and information domains. The highest impact on the model was related to the support domain (impact factor=0.87).

**Conclusion:**

The present pattern is an appropriate and verified Iranian model in the field of structural-psychological empowerment, which is suggested in the cultural context of Iran, especially in medical universities.

## Introduction

The employee empowerment approach is a modern attitude towards human resource management [1]. Nowadays, over 70% of organizations have adopted modern empowerment strategies to maximize the effectiveness of their workforce [2]. Research has indicated that organizations require knowledge, ideas, energy, and creativity of each employee from frontline workers to top managers in the executive suite to succeed in today’s global business environment [1, 2], which is achieved by empowering employees in optimal organizations [2]. The concept of empowerment involves the relinquishment of traditional models, in which managers think, and employees do, and good employees are those who fully comply with whatever the manager states, be it good or bad [1]. In contrast, in modern models, the staff is empowered as enterprise owner without the need to be encouraged, serving the collective interests of the organization without regular supervision by management [2]. Traditional empowerment approaches are not able to make effective and constructive changes in educational, healthcare, and service organizations. Accordingly, it is necessary to design and develop empowerment patterns tailored to specific organizations [3]. In Iran, some patterns have been designed to empower employees. For example*, Farahani et al*. have designed and validated a psychological empowerment pattern based on the relationship between organizational structure and culture in 2015 [4]. *Hadizadeh et al*. also designed and approved a structural empowerment pattern of employee behavior in 2017 [5]. The patterns mentioned above [4, 5] have only addressed one area of ​​psychological or structural empowerment. On the other hand, these patterns have not been specifically designed for the staff of medical universities. Research has shown that structural empowerment cannot be separated from psychological empowerment and that these two types of empowerment and their dimensions are closely interrelated [1]. A broad search by authors showed no pattern to include both aspects (psychological and structural empowerment); therefore, the present study was conducted with the aim of designing a comprehensive structural-psychological empowerment pattern in the staff of medical sciences universities in Tehran.

## Materials and Methods

### Study Design

This study was exploratory research conducted from June 2017 to October 2018. Study protocol was approved by Islamic Azad University of Tehran (Southern branch) with ethics code IR.IUMS.FMD.REC.1396.5097. The research environment included all the medical and non-medical centers affiliated to medical sciences universities of Tehran. Since the number of participants for factor analysis is proportional to the number of tool items that is estimated to be 5-10 subjects for each item [6], the number of participants in this research was calculated to be 430 given the probability of loss. For sampling, firstly, Tehran University of Medical Science, Iran University of Medical Science, and Islamic Azad University were chosen from among medical sciences universities of Tehran using random cluster sampling. Then, the participants were selected from each university through straight random sampling. The inclusion criteria were as follows: employment at the university during the period of research, more than one year of work experience, and willingness to participate in the study. The incompletely filled questionnaires were excluded from the study. Twenty subjects were either unwilling to participate during the study period or did not completely fill the questionnaires and were excluded (95% participation rate). Sampling was done after explaining the objectives of the research and obtaining informed written consent from the staff. The research units were assured in terms of anonymity, secrecy, and respect for their privacy.

### Pattern Design and Test

To design the pattern, the existing researches, literature, patterns, and tools in the field of empowerment were reviewed. Then, the primary pattern of structural-psychological empowerment was designed and confirmed by experts in the field of human resource management. Based on the primary pattern, the primary tool was developed with 31 items and was subject to psychometric analysis. Validity (face, content, and construct validity) and reliability (internal consistency and stability) were used for the psychometric test. The questionnaires were scored according to the five-point Likert scale as follows: strongly agree=5, agree=4, neutral=3, disagree=2, strongly disagree=1.

### Face Validity

A qualitative method (involving 10 staff members and 10 experts) and a quantitative one (including 10 experts) were used to determine the face validity. The participants were interviewed face-to-face, and the items of difficulty, relevancy, and ambiguity were examined and corrected. To determine face validity, a quantitative method of item influence was used, and the acceptable impact score for each expression was considered ≥1.5 [7].

### Content Validity

Twelve experts were surveyed for content coverage, grammar, use of proper expressions, and appropriate location of items to determine the qualitative content validity. Moreover, content validity was quantitatively determined based on comments by 12 experts using content validity ratio (CVR) and content validity index (CVI). According to Lawshe table and Waltz & Bausell CVI, the CVR and CVI were >0.56 and >0.8, respectively [8], and scale-content validity index (S-CVI) was ≥0.9, which were considered to represent an acceptable criterion [9].

### Initial Reliability

The initial reliability (internal consistency of the tool) was evaluated in a pilot study including 30 staff.

### Construct Validity

Exploratory and confirmatory factor analyses were used to determine to construct validity. Kiser-Meyer-Olkin (KMO) sampling adequacy test was conducted with a minimum acceptable value of 0.60 [10] as well as Bartlett’s test of sphericity. Exploratory factor analysis was done using the extraction method of principal component analysis and Varimax rotation. Afterward, the number of factors was determined according to eigenvalue and score plot considered to be the research team viewpoints. The load factor of each question was considered at least 0.4 in factorized and rotated matrices [11]. Standard estimates of path coefficients and fit index were used for confirmatory factor analysis. Chi-square ratio <3, the goodness of fit index (GFI) >0.8, Root mean square error of approximation (RMSEA) index ≤0.9, normed fit index (NFI), inclusive fitness initiative (IFI), and corrected fitness indices (CFI) >0.9 were considered as acceptable criteria in this study [12].

### Reliability and Final Pattern

Internal consistency and stability (test-retest) were used to estimate reliability. The cutoff point of Cronbach’s alpha was considered to be 0.6 [13]. Internal consistency was tested on 410 staff, test-retest method and interclass correlation (ICC) were used to determine stability, and minimum ICC value was considered to be equal to 0.4 [14]. The test-retest of the questionnaire was done on 30 staff in two stages within nearly two weeks, and ICC coefficient was calculated for all the items as well as the whole tool. In conclusion, the final model of structural-psychological empowerment was designed and approved by experts.

### Data Analysis

Data were analyzed using SPSS software version 24 made by USA IBM as well as descriptive (mean, standard deviation, frequency) and inferential statistics (exploratory factor analysis, Cronbach’s alpha, ICC and Pearson correlation coefficient). AMOS software made by USA IBM was also used to verify the items and factors derived from exploratory factor analysis. P<0.05 was considered as the significance level.

## Results

**Figure 1:**
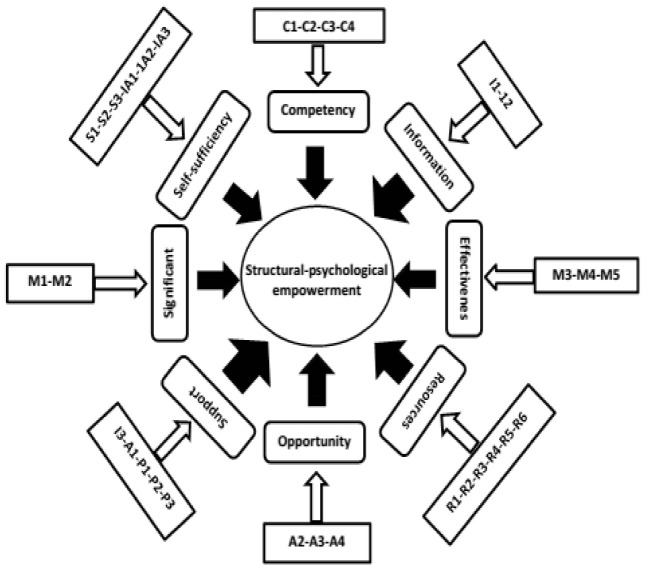
The final model of structural-psychological empowerment

Two hundred ninety out of 410 participants (70.7%) were women. The average ages of the participants were 39.21 ± 5.18 years. A majority of participants was married (70.5%), and most of them had a bachelor’s degree or higher (83.9%). 42.2% of participants had a work experience of fewer than 10 years. Other characteristics of the participants are listed in Table-1. After determining face and content validity in qualitative terms, the items were modified according to the viewpoints of the participants who expressed that the tool was sufficiently comprehensive. All 31 items were preserved in quantitative face validity because of obtaining scores >1.5. CVR and CVI scores of all items and S-CVI/Ave of the tool (0.91) were in an acceptable range (P<0.05). The results of primary tool reliability indicated that the internal consistency of the questionnaire (0.894) and the correlation between scores of each item with the total questionnaire (0.669-0.861) was certified (P<0.05). The results of Bartlett’s test of sphericity (X2=579.874, DF=465, p≤0.05) and KMO test (0.886) were also confirmed. The score plot and eigenvalue specified a total of 8 principal factors, which included 66.77% of the total variance. The names of the factors and load factors of all the items are listed in Table-2. According to standard estimates of path coefficients, the impact of domains was as follows in descending order: support (0.87), information (0.85), opportunity (0.77), resources (0.71), effectiveness (0.52), self-sufficiency (0.50), significance (0.30), and competency (0.23). The significant number of all items in the questionnaire was >2, so all the items were verified. The calculated values ​​of *t* for each factor load were >1.96; therefore, the compatibility of questionnaire items for measurement of concepts was verified (Table-2). The GFI value of 0.748 in the initial pattern was below the permitted limit, which was increased to 0.825 after correction (face, content, and construct validity) and confirmed (reliability). Other indicators of goodness of fit in the primary model as well as after corrections in the final model are presented in Table-3. Based on final reliability, Cronbach’s alpha coefficient and consistency coefficient of the questionnaire were 0.90 and 0.91, respectively (P<0.001). The final model of structural-psychological empowerment was confirmed and designed according to the experts’ opinions as shown in Figure-1.

## Discussion

In the present study, a primary pattern was designed based on a review of existing studies, texts, patterns, and tools in the field of structural-psychological empowerment, which was approved by the research team as well as experts. In this context, some scholars have developed models using previous patterns [15-19], while various scientific sources have been used in the design of the primary model in our research. Similar to our study, *Abesi et al*. [20] and *Fadhil et al*. [21] used various scientific sources to design their own patterns; nevertheless, they designed the patterns with the aim of structural empowerment, while psychological empowerment has been considered along with structural empowerment in the present model. In this research, face and content validity were assessed both quantitatively and quantitatively. In this regard, the researchers state that to determine the face validity of the tool, it is better to use both quantitative and qualitative methods and consider the opinions of target group as well as expert opinion [22]. However, a majority of similar studies [16-18, 20, 21] have not reported face validity in their pattern design. In the researches conducted by *Pahlavansadegh et al.* [15] and *Sadeghi et al.* [19], face validity was reported only qualitatively and on the basis of expert opinion, while in the present study, the viewpoints of staff were reported both the viewpoint of the expert opinion had been reported. The face validity was also quantitatively studied, which is a strong point of our investigation. Both qualitative and quantitative assessments were conducted in this research. In this respect, expert opinion was used, and content validity was confirmed. In line with the results of this study, other similar investigations [16, 17, 19-21] also used qualitative and quantitative methods to assess content validity. Nonetheless, in some other similar studies [15, 18], content validity has not been reported. Therefore, qualitative and quantitative evaluation of content validity is a strong point of our study. To investigate the construct validity, exploratory and confirmatory factor analyses were used in this research. A majority of similar studies have also used both exploratory and confirmatory factor analysis to determine the construct validity of the designed pattern [15, 16-20]. In contrast to the present study, *Fazel et al*. did not use the confirmatory factor analysis [21]. Hence, the use of both types of factor analysis is an advantage of our study relative to the mentioned study. Eight domains were explained in the present study, including resources, self-sufficiency, competence, support, effectiveness, opportunity, significance, and information. The number of domains in similar investigations were as follows: *PahlavanSadiq et al*., three areas of psychological empowerment (self-sufficiency, risk-taking, personal growth) [15], *Abdullahi* five areas of psychological empowerment (competence, self-sufficiency, effectiveness, significance, and trust [17], Fazel *et al.* seven areas of structural empowerment [17], *Hamidizadeh et al.* four areas of structural empowerment (trust, information sharing, education, and reward) [18], *Sadeghi et al*. five areas of psychological empowerment (sense of competence, significance, self-sufficiency, effectiveness, and cooperation with others [19]. There were fewer domains in all these investigations relative to our study perhaps because the study pattern of the present research encompasses both structural and psychological empowerment, while the studies mentioned above have only considered a psychological or structural dimension. The use of both structural and psychological empowerments in the present model is a strong point of our research in relation to the ones mentioned above [15, 17-19, 21]. The highest impact factor in the pattern of the present study was related to support domain. In this domain, the most influential item was “support by managers.” In line with the findings of our study, *Mirmohammadi et al*. showed that support of employees plays a key role in employee empowerment as one of the most important organizational factors, which can increase the sense of trust and train efficient employees [23]. *Amirkabiri et al*. states in this regard that empowerment attempts can backfire in the event of insufficient support from top managers of the organization [24]. Confirmatory validity results in this study showed that the final model of structural-psychological empowerment was appropriate and validated in eight domains. The fitness index results were acceptable in the present research. An appropriate model is the one in which RMR is close to zero, GFI and AGFI are close to 1, RMSEA is less than 0.1, and CFI is higher than 0.9 [18]. The results of pattern fitness index in our research were consistent with the above criteria. The final reliability of the model was approved in our study (α=0.90), and the model had acceptable stability (ICC=0.91). Consistent with this study, other researchers have assessed the reliability of their designed patterns [2, 18, 25]. However, the reliability level of this study was higher than these studies [2, 18, 25]. Unlike the current study, however, some researchers have not reported reliability in the design of model [15-17, 19-21]. Review of reliability and its confirmation in this study is an advantage for it. A major strong point of this study is that the present model is the only one that simultaneously examines the structural and psychological empowerment in Iran. A limitation of this study was that not all medical universities in Tehran were evaluated. Another limitation was the lack of complete filling of some questionnaires (15 questionnaires), which were excluded from the study to resolve this problem.

## Conclusion

This study presented an appropriate and approved Iranian model in the field of structural-psychological empowerment of employees in eight domains. The present model can serve as a guide for improving the structural and psychological empowerment of staff in medical sciences universities in terms of resources, self-sufficiency, competence, support, effectiveness, opportunity, significance, and information. Therefore, it is recommended to use this model in the cultural context of Iran, especially in medical universities.

## Acknowledgment

This paper is derived from Nayereh Sadat Roohollahi’ Ph.D. dissertation (IR.IUMS.FMD.REC.1396.5097) on the management of health services. We would like to thank all the officials, professors, and colleagues who helped us in this research project.

## Conflict of Interest

The authors declared no conflicts of interest.

**Table 1:** Specifications of Participants

**Features**		**Number**	**Percent**
**University**	Tehran	183	44.6
	Iran	177	43.2
	Islamic Azad	50	12.2
**Participation in the training course**	Yes	333	81.2
	No	77	18.8
**Service center**	Hospital	239	58.3
	Faculty	85	2.7
	Administration	86	21
**Employment type**	Formal	219	53.3
	Contractual	22	5.4
	Arbitrary	144	35.2
	Obligatory service	25	6.1
**Work experience**	>10 years	173	42.2
	10-20 years	112	27.4
	>20 years	125	30.4

**Table 2:** Load Factor, Standardized Coefficients, and Significant Number of Structural-Psychological Empowerment Items

**Factor**	**code**	**Items and relevant dimensions**	**Load Factor**	**Standard coefficient**	**Significance number**
**Resources**	R5	My salary is fairly paid	0.825	0.72	13.37
	R6	My reward is fair and performance-based	0.764	0.60	11.19
	R1	I receive the funds (money) I need to do my job	0.74	0.74	13.81
	R4	I am provided with manpower I need to do my job	0.739	0.75	13.9
	R3	I have the time to do my job	0.655	0.65	12.16
	R2	I get the technical equipment I need to do my job	0.648	0.73	-
**Self-sufficiency**	S2	I can decide on how to do a job	0.771	0.70	-
	I1-A	I have a lot of influence on what happens in my unit	0.747	0.70	11.58
	I3-A	My views are taken into account in the decision-making process of the unit	0.73	0.75	12.41
	I2-A	I exert good control over what happens in my unit	0.729	0.73	11.97
	S1	I have the option to do my daily activity	0.617	0.56	17.12
	S3	In the event of a problem, I choose the solution myself and do not have to consult with my superiors	0.601	0.54	9.4
**Competency**	C2	I have mastered the skills needed to do my job	0.807	0.87	-
	C1	I am confident of my ability to do work	0.753	0.79	14.57
	C3	I can solve the problems related to my work without the help of others	0.749	0.56	10.89
	C4	The level of my abilities is higher than the position in which I work	0.716	0.55	10.67
**Support**	P2	I will be supported by my colleagues if necessary	0.807	0.66	10.2
	P3	I will be supported by my subordinate if necessary	0.753	0.62	9.68
	P1	I will be supported by my managers if necessary	0.749	0.81	11.49
	A1	The degree of the authority conferred by my supreme authority satisfies me	0.469	0.62	9.83
	I3	I know the policies and goals of the organization	0.459	0.59	-
**Effectiveness**	M3	I am aware of the goals of organization	0.805	0.74	-
	M5	I agree with the organization’s goals	0.695	0.71	12.54
	M4	I completely understand the organization’s goal	0.661	0.81	13.25
**Opportunity**	A4	I have a chance for in-service training	0.729	0.56	8.84
	A3	I can continue my education	0.712	0.54	8.59
	A2	My organizational post is promoted according to the rules of the organization	0.618	0.73	-
**Significant**	M1	My job is of importance for me	0.829	0.71	4.61
	M2	My job is personally valuable for me	0.787	0.83	-
**Information**	I2	The information I need to do my job is provided to me	0.621	0.77	12.73
	I1	The information provided in the organization is shared to a large extent, so that each person can obtain their required job information as required	0.516	0.75	-

**Table 3:** Characteristics of GFI for Structural-Psychological Empowerment Factors

**Indicator**	**Initial model**	**Final model**	**Permitted level**
**χ** ^2^	3.555	2.839	<3
**GFI**	0.784	0.825	≥0.8
**AGFI**	0.749	0.854	≥0.8
**RMSEA**	0.079	0.067	<0.10
**CFI**	0.802	0.935	≥0.9
**NFI**	0.746	0.901	≥0.9
**IFI**	0.803	0.947	≥0.9

**χ 2:** χ 2 by degree of freedom, **GFI:** Goodness of fit index, **AGFI:** Adjusted goodness of fit index, **RMSEA:** Root mean square error of approximation, **CFI:** Corrected fitness indices, **NFI:** Normed fit index, **IFI:** Inclusive fitness initiative
